# Factors associated with treatment delay among newly diagnosed tuberculosis patients in Dessie city and surroundings, Northern Central Ethiopia: a cross-sectional study

**DOI:** 10.1186/s12889-018-5823-9

**Published:** 2018-07-28

**Authors:** Abdurahaman Seid, Yeshi Metaferia

**Affiliations:** Department of Medical Laboratory Science, College of Medicine and Health Sciences, Wollo University, P.O. Box 1145, Dessie, Ethiopia

**Keywords:** Tuberculosis, Patients’ delay, Health system’s delay, Delay predictors, Ethiopia

## Abstract

**Background:**

Delayed treatment of tuberculosis (TB) cases increases the risk of death and rate of infection in the community. Early diagnosis and initiation of treatment is essential for effective TB control. The aim of this study was to assess length of delays and analyze predictors of treatment delay of newly diagnosed TB patients.

**Methods:**

A cross-sectional study was conducted in Dessie city and surroundings from April1, 2016 to January 30, 2017. Fifteen health facilities of study area were selected randomly and 382 adult TB patients were included consecutively. Data were collected using a questionnaire and analyzed using SPSS version 20.0. Delay was analyzed at three levels (patient, health system and total) using median as cut-off. Logistic regression analysis was performed to investigate predictors of delays. A *p*-value of ≤0.05 at multivariate analysis was considered statistically significant.

**Results:**

The median total, patients’ and health system’s delay was 36 [interquartile range (IQR): 24, 64], 30 (IQR: 15, 60) and 6 (IQR: 4, 8) days, respectively. About 41 and 47% of patients had prolonged patients’ and total delay, respectively. Practicing self-medication [adjusted odds ratio (AOR): 3.0; 95% CI: 1.3–5.6], having more than three family member in the household (AOR: 1.6; 95% CI: 1.02–2.50), older age (≥55 years) (AOR: 2.7; 95% CI: 1.27–5.83), being smear negative pulmonary tuberculosis (AOR: 2.3; 95% CI: 1.25–4.21) and extrapulmonary tuberculosis (AOR: 2.3; 95% CI: 1.28–4.07) were independent predictors of patients’ delay. Initial visit of general practitioners (AOR: 2.57; 95% CI: 1.43–4.63) and more than one health care visit (AOR: 2.12; 95% CI: 1.30–3.46) were independent predictors of health system’s delay. However, patients’ delay was shorter among widowed/divorced patients (AOR: 0.3; 95% CI: 0.1–0.8). Lower level of education [illiterate (AOR: 0.42; 95% CI: 0.20–0.92), grade 1–8 (AOR: 0.38; 95% CI: 0.18–0.81)] and diagnosis of TB using a chest X-ray (AOR, 0.32; 95% CI, 0.16–0.68) significantly reduce health system’s delay.

**Conclusion:**

About half of TB patients delayed beyond 36 days before starting treatment, and the late patient health seeking behavior was the major contributor of total delay. Development and implementation of strategies aimed at addressing identified factors should be recognized in order to reduce TB treatment delay. Further well designed research is needed to explore additional risk factors of delayed treatment.

## Background

Tuberculosis (TB), caused by *Mycobacterium tuberculosis*, is an infectious disease which has a major global public health implications [1, 2]. In 2015, an estimated 10.4 million new (incident) TB cases and 1.8 million TB deaths were reported worldwide [1], which is much higher than 2013 estimates [2]. Even though important diagnostic and treatment gaps remain to be addressed, TB treatment prevented 49 million deaths globally between the years 2000 to 2015. According to the world health organization (WHO) report, six countries such as India, Indonesia, China, Nigeria, Pakistan and South Africa in decreasing order accounted for 60% of new TB cases in 2015. Ethiopia stands 10th of the thirty high TB burden countries of 2015 [1] and the country didn’t show any improvement from previous WHO report [2].

Globally, effective TB control depends on major improvement in TB prevention and care strategies in these high burden countries including Ethiopia [1]. Thus, early diagnosis of infectious TB cases and treating them effectively are the keystones of global TB control programs [3]. However, late diagnosis and treatment of TB cases have been identified as a major obstacle of TB control program especially in low income countries like Ethiopia. Prolonged treatment delay increases the risk of spreading infection and out-of-pocket expenditure of patients in the community, limits the success of TB treatment and is accompanied by a high risk of morbidity and mortality [4–6]. For instance, an untreated smear positive TB case become source of infection for about 10 contacts annually and over 20 during the natural history of the disease until death occurs [7, 8]. Components of different delay periods staring from the onset of TB symptom to the commencement of treatment were related to patient, health care system or a combination of both. Different factors contributing for delayed treatment of TB patients have been assessed across the globe and broadly classified as factors associated to patient, health care provider or a combination of the two [7–10].

In Ethiopia, various studies revealed that treatment delay is still one of the fundamental TB control problems contributing to the observed high TB burden in the country [6, 9, 11–15]. Studies conducted among pulmonary tuberculosis (PTB) patients indicated that the median patients’ delay was ranged from 20 to 30 days [5, 10, 14] and health system’s delay was ranged from 33.5 to 42 days [5, 10]. Similarly, studies conducted among PTB and extra pulmonary tuberculosis (EPTB) patients indicated a comparable median patients’ delay of 21 to 30 days [16, 17], but lower health system’s delay (6 to 27 days) [12, 17, 18]. PTB patients with smear positive status are known to be the significant source of infection to the community when compared to other types of TB patients [19]. Besides recognized as significant infectious pool to the community, smear positive PTB patients experienced a median total delay of 35 to 80 days [9, 15, 20], patients’ delay of 30 days and health system’s delay of 11 to 21 days [9, 15].

Different factors have been identified as a potential cause of TB treatment delay. The most commonly reported factors are sputum smear negative status, being EPTB patient, living in rural settings; initial visit of a government low-level health care facilities, private practitioners, or traditional healers; and low level of awareness about TB [7]. Despite large number of studies being conducted about the cause of late treatment of TB patients in Ethiopia and elsewhere in the world, relation between longer treatment delay and treatment outcome is not investigated. Furthermore, TB diagnosis and treatment delay period and contributing factors vary among societies, type of visited health facilities and geographical locations; between population groups in the same local settings and category of the disease. These calls for localized studies intended to identify area and population specific factors associated with prolonged delay of TB diagnosis and treatment. The aim of this study is, therefore, to estimate patients’, health system’s and total delay days; and to investigate factors associated with these delay periods among all forms of newly diagnosed TB patients in Dessie city and surroundings.

## Methods

### Study setting

The study was conducted in Dessie city and surrounding woredas such as Kalu, Tehweldre, Kutaber, Dessie zuriea, and Albuico, which are located in South Wollo, Ethiopia. Dessie is one of the major cities found in Amhara region, northern central Ethiopia. Dessie is located at 401 km from the capital of Ethiopia, Addis Ababa. In the study area there were 53 public health facilities (one referral hospital, one general hospital, and fifty one health centers), three private hospitals and thirty eight private clinics during the study period. In the study area patients have had different options to seek health care. In Ethiopian health care delivery system health post is the initial and low level of health care. It is available in each Kebele (the lowest administrative unit of the country with an average population of 5000) and is assisted by at least one female health extension professionals who are trained for one year in basic preventive and promotive health services. Moreover, in Ethiopia, all public health facilities provide free of charge health services for TB patients through a network of hospitals, health centers, clinics and health posts. However, TB diagnosis is not performed in the health posts.

### Study design and population

A health facility based cross sectional study was conducted from April1, 2016 to January 30, 2017. The study population consisted of all forms of newly diagnosed adult TB patients who were registered to the selected health facilities during the study period. Sample size was determined using single population proportion formula as follows. N = [(Zα/2)^2^ ∗ p ∗ (1-p)] /d^2^ where: N = total sample size, Zα/2 at 95% CI is 1.96, d marginal error 0.05, and **P** is the proportion (47.8%) of TB patients who started treatment after 60 days (median total delay) of initial onset of signs and symptoms [17]. The calculated sample size was 384. The study was carried out in 15 randomly selected public health facilities that provide directly observed treatment short course (DOTS) in the study area. Total sample size was proportionately allocated for the 15 randomly selected health facilities based on their previous one year estimated number of registered TB patients. The proportions of participant TB patients were as follows: 35 from one referral hospital, 18 from one general hospital and 337 from 13 health centers**.** Thus, TB patients who fulfilled the inclusion criteria were included consecutively from the above mentioned public health facilities until the calculated sample size was attained.

New patients (≥18 years old) diagnosed, based on the national TB guideline [21], with TB of all forms and who had commenced treatment for less than 15 days at the time of data collection were included in the study. TB patients transferred to the selected health facilities for continuation of treatment were included if the transfer was within 15 days of starting treatment. Double counting of study participants was avoided through attachment of reminder note with referral sheet. However, TB patients who were relapsed or treatment failed, critically ill, unable to communicate, refused to participate and to give consent for human immunodeficiency virus (HIV) screening were excluded. TB patients who were temporary residents of the study area (living for < 6 months) were also excluded from the study.

### Operational definitions of variables

Different delay periods were defined similarly to previous studies, and median value was used as a cut-off value to make simple comparison with previous similar studies.

Patient delay: the time interval (in days) between the initial onsets of the first symptoms of TB until the first visit to a formal health care provider. TB patients who consulted a formal health care provider longer than the median value after the onset of the initial constitutional signs and symptoms of TB were considered delayed [10, 17].

Health system delay: the time (in days) interval from patient’s first visit of formal health care provider to commencement of anti-TB treatment. This may comprise of days spent for TB diagnosis and referrals. If the time after initial reporting to a formal health care provider to initiation of treatment is more than the median value it is considered as ‘delayed’ [10, 17].

Total delay: The time interval (in days) between the onsets of the main TB symptoms, reported by the patient as his/her chief complaints, to initiation of anti TB treatment. Thus, period more than the median value was considered as ‘delayed’ [10, 17].

Formal-health care providers: modern government or private health care facilities such as clinics, health centers and hospitals [10, 17].

Self-medication: any kind of self-prescribed or home prepared medications taken by patients for the treatment of TB.

New TB patient: is a confirmed TB patient who has never had TB disease before.

Illiterate: TB patients with no formal education and unable to read and write.

The different category of TB patients is based on the national TB control guideline [21], and operationalized as follow:

Pulmonary Tuberculosis: a patient who develops tuberculosis that exclusively affect the lung parenchyma.

Smear-positive PTB: a patient with at least two initial smear-positive sputum examinations for acid fast bacilli (AFB), or one initial smear-positive examination by direct microscopy and culture positive, or one initial smear-positive examination and radiographic findings consistent with active TB as determined by a clinician.

Smear-negative PTB: a patient with at least three initial smear-negative sputum examinations for AFB and no response to broad-spectrum antibiotics, or three smear-negative examinations by direct microscopy and radiological findings consistent with PTB and a decision by a physician to treat with a full course of anti-TB drugs, or when a patient’s diagnosis is based on culture-positive *Mycobacterium tuberculosis* despite three initial smear-negative examinations by direct microscopy.

Extrapulmonary tuberculosis: a patient with TB in organs other than the lungs and indicated by one culture-positive specimen from an extrapulmonary site or histopathological evidence from a biopsy, or strong clinical evidence suggestive of active EPTB and the decision by a physician to start treatment with anti-TB chemotherapy.

Multiple healthcare visits: this is defined as consulting of a health care facility two or more times, irrespective of their type as government or private, before TB diagnosis confirmation is made.

### Instrument and data collection

A standardized questionnaire, adapted from a previous validated WHO questionnaire [22], was used for the collection of socio-demographic, clinical and other related information. Initially, English version questionnaire was prepared and then translated to the local “Amharic version”, and again back translation to English version was made to ensure the consistency of the question. The final questionnaire was prepared in “Amharic version” and pre-tested on 10 new TB patients who started their treatment in a public health facility that is not included in the current study, and important modifications that need to be incorporated was made in the final version. A One day training was given for nurses and/or health officers (data collectors) working at selected health facilities about the contents of the questionnaire and strategy of interviewing the study participants.

After getting informed consent, data collectors interviewed the participants to collect information like residence, education level, occupation, marital status, family size, practice of self-treatment and use of nonprescribed drugs for TB treatment, time required to reach a health facility, previous exposure to TB patient, the date when TB symptoms started and initial visit of the TB patient to health care provider, health facility visiting frequency and types of health facility, the time when TB diagnosis is confirmed, and other variables of interest. The date of initial start of main symptoms was taken as the date of onset for the illness. Cough was considered as the main symptom of PTB patients whereas either of localizing symptoms like swelling lymph nodes for TB lymphadenitis, chest pain for TB pleurisy or constitutional symptoms (fever, night sweat, weight loss and loss of appetite) was considered as the initial onset of EPTB. Patient’s recall ability was supported by using a calendar of locally important events such as main religious days, national holy days and other probing techniques [17].

National HIV test algorithm was employed for screening of TB patients. It was done using HIV (1 + 2) Antibody Colloidal Gold (KHB, Shanghai Kehua Bio-engineering Co Ltd., China) as a screening test. KHB reactive TB patient was tested by HIV ½ STAT-PAK® (Chembio Diagnostics, USA). Discordant result of STAT-PAK® and KHB was determined and confirmed by a tiebreaker, Unigold™ HIV (Trinity Biotech, Ireland). Additionally, age and sex of TB patients, chief TB symptoms and its initial date of onset, date of first diagnosis of current illness and specialty of the health care provider who made the initial diagnosis, laboratory methods used for TB diagnosis and date of initiation of anti TB-treatment were collected and cross-checked from patient laboratory records, patient register cards, and patient files. Weekly supervision of data collection was made by the researchers and the TB program officer of each health facility.

### Data analysis

The collected data were entered into Epi-data version 3.5.3 and exported to SPSS software version 20.0 for analysis. Frequency distributions, mean, median and interquartile range (IQR) were computed. The normality of different delay periods was checked using Shapiro test and all delay periods are not normally distributed. Group comparisons were made using the t-test for means, chi-square test (χ2) for proportions, Mann–Whitney and Kruskal-Wallis test for median of two and more than two groups, respectively. Most Ethiopian studies used median value as an acceptable cut-off for delay periods [6, 9, 10, 16, 18], and thus we adopted the median value to categorized TB patients into delayed and not delayed.

Logistic regression analysis was performed to assess factors associated with the different delay periods. Bivariate analysis was performed on each variable and a respective crude odd ratio (COR) was calculated. Confounders were controlled by running stepwise backward logistic regression analysis. Independent variables with marginal associations (*P* ≤ 0.20) in the bivariate analysis were entered in a multivariate logistic regression analysis in order to detect independent predictors of patients’ delay (> 30 days), health system’s delay (> 6 days) and total delay (> 36 days). The goodness of fit of utilized model was assessed using the Hosmer-Lemeshow test. The significant association of independent variables with the dependent variable was assessed by using 95% confidence interval (CI) and a respective adjusted odds ratio (AOR). A two tailed-sided *p* value of ≤0.05 was taken as statistically significant.

## Results

### Study participant sociodemographic characteristics

During the study period 390 TB patients were enrolled from randomly selected 15 DOTS providing public health facilities, but 8 patients were excluded from analysis due to incomplete information on TB registration books and questionnaire. Thus, data of 382 TB patients were analyzed. The socio-demographic profile and clinical characteristics of study participants are shown in Tables 1 and 2 respectively, and their distribution with different delay period is shown in Table 3. The mean age of study participants was 37.14 years (median 33.5, IQR 25–48), ranged from 18 to 88 years. From the total study participants, 52.6% were male, 42.9% were in the age group 26 to 44 years, 72% were urban residents, 56.8% were married, 30.4% were farmer in occupation, and 35.6% had a primary education level (grade 1–8). The median number of household members was 3 (IQR 2–4). The median one way time required by patients from their residence to initially visited health facility was 30 min (IQR 20–60) with a mean of 46.47 min (Table 1).

**Table 1:** Socio-demographic characteristics of TB patients in Dessie city and surrounding, Ethiopia, 2016 (*n* = 382)

Characteristics		n (%)
Sex		
Male		201 (52.6)
Female		181 (47.4)
Residence		
Urban		275 (72)
Rural		107 (28)
Age (in years)		
18–25		107 (28)
26–44		164 (42.9)
45–54		46 (12)
≥ 55		65 (17)
Education		
Illiterate		115 (30.1)
grade 1–8		136 (35.6)
grade 9–12		89 (23.3)
college and above		42 (11)
Occupation		
farming		116 (30.4)
business		63 (16.5)
student		52 (13.6)
unemployed		66 (17.3)
employed		85 (22.3)
Marital status		
married		217 (56.8)
never married		133 (34.8)
widowed/divorced		32 (8.4)
Family size		
1 to 3		208 (54.5)
> 3		174 (45.5)
One way walking time	≤ 30 min	226 (59.2)
	> 30 min	156 (40.8)

**Table 2:** Clinical characteristics and health seeking behavior of TB patients in Dessie city and surrounding, Ethiopia, 2016 (*n* = 382)

Characteristics		n (%)
Practiced self-medication	No	326 (85.3)
	Yes	56 (14.7)
Use of non-prescribed medications	No	348 (91.1)
	Yes	34 (8.9)
TB category	SPPTB	96 (25.1)
	SNPTB	132 (34.6)
	EPTB	154 (40.3)
Exposure to TB patient	Yes	39 (10.2)
	No	343 (89.8)
HIV serostatus	Positive	78 (20.4)
	Negative	304 (79.6)
Initially visited HF	Gov’t	301 (78.8)
	Private	81 (21.2)
Diagnostic tests	microscopy	59 (15.4)
	Chest X-ray	109 (28.5)
	microscopy and chest X-ray	179 (46.9)
	Gene-Xpert	35 (9.2)
Initially consulted HCP	Internist	108 (28.3)
	GP	159 (41.6)
	Nurses/HO	115 (30.1)
Health care contacts	Single	149 (39)
	Multiple	233 (61)

*HCP* health care provider, *GP* general practitioner, *HO* health officer, *HF* health facilities

**Table 3:** Distribution of diagnostic delay by socio-demographic, clinical variables and health seeking trajectories, nonparametric (Mann–Whitney and Kruskal-Wallis) test

Characteristics	Patient delay		Health System delay		Total delay	
	Median (IQR)	*p*-value	Median (IQR)	*p*-value	Median (IQR)	*p*-value
Total	30(15,60)		6(4,8)		36(24,64)	
Delayed, n (%)	157(41.1)		134(35.1)		181(47.4)	
Sex						
Male	30(15,60)	0.771	6(4,8)	0.063	35(22.5,64)	0.899
Female	30(15,60)		6(4,7)		36(24.5,63)	
Residence						
Urban	30(15,50)	**0.025**	5(4,7)	**0.009**	35(21,58)	0.008
Rural	31(20,60)		6(5,9)		40(28,65)	
Age (years)						
18–25	30 (15,45)		6(4,8)		34(21,53)	
26–44	30 (15,60)	0.109	6(4,7)	0.704	36(26,64)	0.157
45–54	30 (15,52.5)		6(5,7)		34.5(20,57.5)	
≥ 55	40 (20,60)		6(4,7)		49(26.5,65)	
Education						
Illiterate	30(15,60)	0.231	6(4,7)	0.070	39 (25,64)	0.362
grade 1–8	30(15,60)		6(4,7)		36 (25,63)	
grade 9–12	30(15,45)		6(4,8)		35 (21.5,50)	
college &above	23.5(15,45)		6.5(5,9)		30.5 (22.5,59.3)	
Occupation						
farming	32.5(20.25,60)	**0.033**	6(4.25,9)	0.070	45(28,68)	**0.010**
business	30(15,45)		5(4,6)		34(21,56)	
student	30(15,40)		6(5,8)		35(21,44.75)	
unemployed	30(15,45)		6(4,7)		34.5(21,49)	
employed	30(15,60)		6(4,8)		35(22.5,64)	
Marital status						
married	30(15,60)	**0.015**	6(4,7)	0.069	37(25,64)	**0.006**
never married	30(15,60)		5(4,8)		35(24,64)	
widowed/divorced	18(15,30)		5(4,6)		26(19,40.25)	
Family size						
1 to 3	30(15,57.5)	**0.011**	6(4,7)	0.076	34(21,60.5)	**0.002**
> 3	30(20,60)		6(5,8)		40(27.75,60)	
Self-medication						
No	30(15,47.75)	**0.000**	6(4,8)	0.990	35(22,56.25)	**0.000**
Yes	50(30,60)		6(4,8)		56(34.25,70.75)	
Use of non-prescribed drugs						
No	30(15,60)	**0.005**	6(4,8)	0.073	35(22,63)	**0.006**
Yes	37.5(30,62.5)		5(4,7)		44(34,72.75)	
One way walking time						
≤ 30 min	30(15,60)	**0.004**	5(4,7)	**0.001**	34(20.75,63)	**0.000**
> 30 min	30(20,60)		6(5,8)		40.5(28.25,64)	
TB category						
SPPTB	30(15,40)	0.145	6(5,7)	0.843	34(24.25,48)	**0.037**
SNPTB	30(15,60)		5.5(4,7)		35(21,64)	
EPTB	30(15,60)		6(4,8)		41.5(27.75,64)	
Previous exposure to TB patient						
Yes	34(19,60)	**0.049**	6(4,8)	0.539	45(27,68)	**0.043**
No	30(15,58)		6(4,8)		35(23,63)	
HIV serostatus						
positive	30(15,45)	0.287	5(4,7.25)	0.129	34.5(21,54.5)	0.197
Negative	30(15,60)		6(4,8)		36(25,64	
Initially visited health facilities						
Government			6(4,7)	0.079	37(26,64)	0.041
Private			6(5,8)		30(21,57.5)	
Diagnostic tests						
microscopy			6(4,9)	**0.000**	29(20,41)	**0.000**
Chest X-ray			5(4,6)		30(19,49)	
microscopy and chest X-ray			6(5,8)		39(28,65)	
Gene-Xpert			7(5,12)		52(30,67)	
Initially visited HCP						
Internist			5(4,6.75)	0.104	21.5(19,35)	**0.000**
General practitioners			6(4,8)		41(29,65)	
Nurse/health officer			6(4,7)		42(29,65)	
Health care contacts						
Single			5(4,7)	**0.003**	33(21,46.5)	**0.000**
Multiple			6(5,8)		38(26,65)	

*HCP* health care providers, *IQR* interquartile range, *SPPTB* smear positive pulmonary tuberculosis, *SNPTB* smear negative pulmonary tuberculosis, *EPTB* extrapulmonary tuberculosis

Statistically significant value are in bold

### TB patients health care seeking behavior

Out of the total participants, 10.2% had previous exposure to TB patients, 14.7% used different forms of home prepared self-medication, and 8.9% used different non-prescribed medications before visiting formal health care provider. Ninety six (25.1%) and 132 (34.6%) participants were smear-positive PTB (SPPTB) and smear-negative PTB (SNPTB) cases, respectively. After the initial onset of TB signs and symptoms 78.8% of participants visited public health care providers and 41.6% consulted general practitioners (GP). Nearly 2/3rd of TB patients had multiple healthcare contacts before diagnosis confirmation and 46.9% were diagnosed using a combination of chest X-ray and microscopy (Table 2).

### Delay period and determinants

The overall median time of patients’, health system’s and total delay was 30 days (IQR 15, 60), 6 days (IQR 4, 8) and 36 days (IQR 24, 64), respectively. Group comparison of variables was made using Mann–Whitney and Kruskal-Wallis test. For instance, median patients’ delay did not show significant variation among SPPTB, SNPTB, and EPTB patients (Kruskal-Wallis, *P* > 0.05). However, significantly prolonged patients’ delay was found among rural dwellers, those who practiced self-medication and use nonprescribed drugs at home, and those who previously exposed to TB patients (Mann-Whitney, *p* < 0.05). Significantly decreased patients’ delay was also observed among widowed/divorced participants (Mann-Whitney, *p* < 0.05). The distribution of different delay periods in relation to socio-demographic and other variables is shown in Table 3. In all, 157 (41.1%), 134(35.1%) and 181(47.4%) patients had prolonged (above the median) patients’, health system’s and total delay, respectively. Similarly, 30.2% (29), 43.2% (57) and 46.1% (71) of SPPTB, SNPTB and EPTB patients consulted a health care provider beyond 30 days of onset of symptoms, respectively.

### Determinants of patients’ delay

On bivariate logistic regression, prolonged patients’ delay was significantly associated with rural residence, older age (≥55 years), having more than three family members in the household, practicing self-medication, walking for more than 30 min to reach a health facility, smear-negative status and EPTB. But divorced/widowed marital status was significantly associated with reduced patients’ delay. On multivariate logistic regression, independent determinants of longer patients’ delay were older age (≥ 55 years) (AOR: 2.7; 95% CI: 1.27–5.83), large family members in the household (AOR: 1.6; 95% CI: 1.02–2.50), practicing self-medication (AOR: 3.0; 95% CI: 1.3–5.6), SNPTB (AOR: 2.3; 95% CI: 1.25–4.21) and EPTB (AOR: 2.3; 95% CI: 1.28–4.07). Widowed/divorced marital status was independent predictors of reduced patients’ delay (AOR: 0.3; 95% CI: 0.1–0.8) (Table 4).

**Table 4:** Factors associated with patients’, health system’s and total delay for treatment of TB patients, Dessie city and surroundings, bivariate and multivariate analysis, 2016

Characteristics	Patient delay > 30 days		Health System delay > 6 days		Total delay > 36 days	
	COR (95% CI)	AOR(95% CI)	COR (95% CI)	AOR(95% CI)	COR (95% CI)	AOR(95% CI
Sex						
Male	1		1		1	
Female	1.2(0.8–1.8)	_	0.6(0.42–0.98)•	_	1.1(0.7–1.6)	_
Residence						
Urban	1		1		1	
Rural	1.7(1.1–2.7)•	1.58(0.95–2.62)	1.3(0.8–2.0)	_	1.5(0.9–2.3)*	_
Age (years)						
18–25	1		1		1	
26–44	1.4(0.8–2.3)	1.7(0.93–3.06)	0.8(0.5–1.3)	_	1.1(0.7–1.8)	_
45–54	1.3(0.6–2.6)	1.4(0.62–3.25)	0.7(0.3–1.4)	_	0.7(0.4–1.5)	_
≥ 55	2.5(1.3–4.6)•	2.7(1.27–5.83)•	0.7(0.3–1.3)	_	1.84(0.99–3.45)*	_
Education						
Illiterate	1.5(0.8–3.2)	_	0.5(0.2–1.0)*	0.42(0.20–0.92)•	1.5(0.7–3.1)	_
Grade 1–8	1.1(0.6–2.3)	_	0.5(0.2–0.9)•	0.38(0.18–0.81)•	1.3(0.7–2.6)	_
Grade 9–12	0.8(0.4–1.7)	_	0.6(0.3–1.2)*	0.59(0.27–1.31)	0.9(0.4–1.8)	_
College &above	1		1	1	1	
Occupation						
farming	1.3(0.8–2.4)	_	1.1(0.62–1.97)	_	1.75(0.99–3.08)*	_
business	0.7(0.3–1.3)	_	0.5(0.21–0.96)•	_	0.8(0.3–1.5)	_
student	0.6(0.3–1.3)		1.4(0.7–2.8)	_	0.98(0.49–1.97)	_
unemployed	0.7(0.4–1.4)	_	0.9(0.4–1.7)	_	0.9(0.5–1.7)	_
employed	1		1		1	
Marital status						
married	1	1	1		1	
never married	0.8(0.5–1.2)	1.3(0.7–2.2)	1.0(0.7–1.6)	_	0.7(0.5–1.1)*	_
widow/divorce	0.3(0.1–0.7)•	0.3(0.1–0.8)•	0.5(0.2–1.2)*	_	0.4(0.2–0.8)•	_
Family size						
1 to 3	1		1		1	1
> 3	1.6(1.1–2.4)•	1.6(1.02–2.5)•	1.3(0.8–1.9)	_	1.9(1.3–2.9)•	1.7(1.1–2.8)•
Self-treatment						
No	1	1	1		1	1
Yes	2.8(1.6–5.0)•	3.0(1.3–5.6)•	1.0(0.6–1.9)	_	2.97(1.62–5.47)•	2.03(1.001–4)†
Use nonprescribed drugs						
No	1		1		1	
Yes	1.9(0.95–3.92)*	_	0.8(0.4–1.6)	_	1.7(0.8–3.4)*	_
One way walking time						
≤ 30 min	1		1		1	1
> 30 min	1.7(1.1–2.6)•	_	1.6(1.1–2.5)•	_	2.2(1.5--3.4)•	1.5(0.92–2.52)
TB category						
SPPTB	1		1		1	1
SNPTB	1.8(1.01–3.06)•	2.3(1.25–4.21)•	0.99(0.6–1.7)	_	1.4(0.8–2.4)	2.1(1.08–4.08)•
EPTB	1.98(1.2–3.4)•	2.3(1.28–4.07)•	1.1(0.6–1.9)	_	2.3(1.3–3.8)•	3.2(1.62–6.19)•
Previous exposure to TB patient						
Yes	1.8(0.9–3.5)*	_	1.5(0.8–2.9)	_	1.7(0.9–3.3)*	_
No	1		1		1	
HIV sero-status						
positive	0.8(0.45–1.27)	_	0.7(0.39–1.16)	_	0.8(0.50–1.36)	_
negative	1		1		1	
Initially visited health facilities						
Government			1		1	1
Private			1.4(0.82–2.25)	_	0.6(0.33–0.91)•	0.5(0.26–0.99)•
Type of lab diagnosis						
Microscopy			1	1	1	1
Chest X-ray			0.3(0.2–0.6)•	0.32(0.16–0.68)•	1.2(0.6–2.3)	1.18(0.5–2.8)
Microscopy & chest X-ray			0.8(0.4–1.4)	0.56(0.29–1.06)	2.95(1.57–5.53)•	2.5(1.17–5.36)•
Gene-Xpert			1.5(0.7–3.5)	1.21(0.49–2.93)	5.69(2.27–14.28)•	4.4(1.5–13.5)•
Initially visited HCP						
Internist			1	1	1	1
General practitioners			2.2(1.3–3.7)•	2.57(1.4–4.6)•	5.6(3.2–9.9)•	4.4(2.2–8.6)•
Nurse/health officer			1.6(0.90–2.9)*	1.58(0.84–2.95)	4.3(2.4–7.8)•	3.2(1.5–6.6)•
Health care contacts						
Single			1		1	
Multiple			2.1(1.3–3.2)•	2.12(1.30–3.46)•	1.9(1.3–2.9)•	2.4(1.4–4.1)•

•*p* < 0.05; *p ≤ 0.2; †*p* = 0.050

*HCP* health care providers, *SPPTB* smear positive pulmonary tuberculosis, *SNPTB* smear negative pulmonary tuberculosis, *EPTB* extrapulmonary tuberculosis

### Determinants of health system’s delay

On bivariate logistic regression, prolonged health system’s delay was significantly associated with greater walking time (> 30 min) to reach a health facility, initial consulting of general practitioners and multiple healthcare contacts. TB patients with female gender, primary level of education (grade 1–8) and business as major occupation were less likely to have prolonged health system’s delay. Furthermore, diagnosis of TB using chest X-ray was more likely to reduce health system’s delay. On multivariate analysis, only lower level of educational status [illiterate (AOR: 0.42; 95% CI: 0.20–0.92), grade 1–8 (AOR: 0.38; 95% CI: 0.18–0.81)] and doing a chest X-ray for TB diagnosis (AOR: 0.32; 95% CI: 0.16–0.68) were significantly associated with reduced health system’s delay. However, initial visiting of general practitioners (AOR: 2.57; 95% CI: 1.43–4.63) and multiple healthcare contacts (AOR: 2.12; 95% CI: 1.30–3.46) were found to be independent determinants of longer health system’s delay (Table 4).

### Determinants of prolonged total delay

Analysis result of bivariate logistic regression indicated that prolonged total delay was significantly associated with large family member in the household, practicing self-medication, longer walking time (> 30 min) to reach a health facility, EPTB, initial consulting of general practitioners and nurses/health officers. Multiple healthcare contacts, use of gene-Xpert and a combination of microscopy and chest X-ray were also significantly associated with prolonged total delay. Initial visiting of a private health facility was found to significantly reduce total delay as compared to a government health facility. Widowed/divorced marital status was also significantly associated with shorter total delay (Table 4).

On multivariable analysis, having large family size (AOR: 1.7; 95% CI: 1.1–2.8), SNPTB (AOR: 2.1; 95% CI: 1.08–4.08), EPTB (AOR: 3.2; 95% CI: 1.62–6.19), initial visiting of general practitioners (AOR: 4.4; 95% CI: 2.2–8.6) and nurses/health officers (AOR: 3.2; 95% CI: 1.5–6.6), multiple healthcare contacts (AOR: 2.4; 95% CI: 1.4–4.1), use of gene-Xpert (AOR: 4.4; 95% CI: 1.5–13.5), in combination use of microscopy and chest X-ray (AOR: 2.5; 95% CI: 1.17–5.36) were independent predictors of longer total delay. However, initial visiting of a private health facility reduces total delay by half as compared to visiting a government health facility (AOR: 0.5; 95% CI, 0.26–0.99) (Table 4).

## Discussion

In the current study, total treatment delay is majorly contributed by the late patient health seeking behavior than health system’s delay, which is similar to previous reports from Africa [6, 23]. The observed less contribution of health system’s delay might be due to the recent progressive improvement of health facilities in Ethiopia. However, our finding suggests a need of improving patients’ health seeking behavior through awareness creation about importance of early recognizing TB symptoms.

In our study, the observed 30 days of overall median patient delay is comparable with previous studies in Ethiopia (28–30 days) [16, 24–26] and other low and middle income countries (31.7 days) [27]. But, higher (60–63 days) [6, 12, 18] and lower (20–21 days) [10, 17, 28] median patient delay period were documented in Ethiopia. Lower median patient delay (14 days) was also reported in France [29]. The observed variation might be due to differences in study period, study area health system infrastructure, patients’ socio-demographic characteristics, level of patients’ knowledge about TB, and lack of awareness about disease symptoms and free availability of treatment. In our study, prolonged patients’ delay might be also due to patient’s inability to recognize symptoms at early onset of the disease. This is supported by findings of a recent TB prevalence survey where less than half of the TB cases fulfill the criteria of being a “TB suspect” and many have only vague symptoms [30].

In the current study, the proportion of patients who delayed beyond the median value of patients’ delay (30 days) was 41.1%. Even though there is variation of median patients’ delay days, the observed proportion was comparable with a previous report in Ethiopia (43.8%) [18]. However, lower (24–36.7%) [10, 17, 26] and higher (52.4%) [16] proportion of TB patients reported to health care provider beyond the median period (30 days). Practicing self-medication was found to be an independent predictor of patients’ and total delay which is consistent with previous reports from Ethiopia [9, 10, 26]. In developing countries, about half of the general population uses prolonged traditional healing systems as the first step in the health-seeking process [31, 32]. This suggests a need of encouraging TB symptomatic individuals to seek the appropriate modern health care provider as early as possible rather than visiting traditional healthcare providers.

In our study, patient residence and sex had no significant association with patients’ delay which is consistent with other reports [12, 26], but it is in opposition to previous reports in Ethiopia where women were less likely to have appropriate health-seeking behavior than men [12, 17, 24]. The observed residence’s lack of association might be due to improved access of health care services to both the rural and urban community. Moreover, recent gender equality practice in Ethiopia also empowered women to decide on their own health issues and participate in TB awareness creation and promotion activities in the country.

Older patients (≥55 years) had an increased risk of prolonged patients’ delay which is comparable with a previous study [17], but contrary to other reports with no significant association between patient delay and age [11, 12]. The observed significant association might be due to the fact that older patients are dependent on others to seek health care when needed. Moreover, in older patients TB disease may not show typical symptoms indicative of TB rather the clinical presentation are nonspecific [33], which leads to a low index of TB suspicion by health care providers. However, signs and symptoms of TB that are specifically associated with older patients should be investigated.

Previous reports illustrated that illiterate or lower level of education was found to be predictor of prolonged TB treatment delay [6, 16, 25] which is against to our findings with lower level of education (below grade 9) more likely to reduce health system’s delay (Table 4). Even though lower levels of education indicate poor TB knowledge which indirectly increases delay time, recent evidence suggested that TB treatment initiation is more influenced by perceptions than knowledge [34]. Thus, good perception of TB patients with lower educational status might be the possible justification for the currently observed association. However, the level of population perception and its effect on treatment delay needs to be explored. In our study, widowed/divorced patients were 0.3 times less likely to have prolonged patients’ delay as compared to married ones. This might be due to health care awareness through previous diseases since 46.9% widowed/divorced participants were HIV seropositive. Nevertheless, this warrants further study to document the impact of being widowed/ divorced on TB control program.

Larger family size contributes for prolonged patients’ and total delay in this study. This could be due to the fact that those who had larger family members shouldered more family responsibilities and experienced severe socioeconomic hardships, which prevented them from timely visiting appropriate healthcare facilities for their illnesses. The site of infection and smear status was also significant predictor of patients’ delay. EPTB and SNPTB patients were more delayed to seek health care which is parallel to other studies in Ethiopia [12, 16] where EPTB was identified as independent predictors of patients’ delay. Smear negative status and EPTB also contributed for prolonged total delays which is similar to the findings of other studies [17, 35]. EPTB patients may have a variety of symptoms, which could easily be overlooked and leads to delayed health seeking behavior. It is also likely that many of the EPTB cases might have practiced self-treatment, which is a significant predictor of prolonged patients’ and total delay in our study. Health centers of the study area have no facilities for diagnosis of EPTB and SNPTB cases. Besides this fact, it is documented that definitive diagnosis of SNPTB is more difficult than smear positive counter parts, making the treatment initiation delayed for a longer period of time [7].

The overall median health system’s delay was 6 days which is consistent with recent reports in Ethiopia, 5 to 6 days [18, 36], but lower than previous reports in Ethiopia, 9 to 15 days [6, 12, 26], France, 25 days [29] and Taiwan, 29 days [37]. In this study, about 35.1% of patients encountered the health system’s delay which is lower than reports of Hamza et al. [26] in Arsi, Ethiopia, with 49.7% patients experienced health systems’ delay. Similarly, we observed the overall median total delay of 36 days, which is lower than previous studies conducted in Ethiopia, 40–75 days [6, 10, 17, 18, 26] and France (68 days) [29]. The lower observed health system’s and total delay in relative to other mentioned studies [6, 10, 12, 17, 18, 26, 29, 37] might be related to recently improved health systems and access to TB care through implementation of the health extension program in the study area.

In our study, multiple healthcare visits, irrespective of their type, significantly increases the health system’s and total delay. It is documented that the problem of delay in diagnosis and treatment of TB is a vicious cycle of repeated visits at the same health care level [7]. Initial visiting of a general practitioner was also identified as a significant predictor of prolonged health system’s and total delay. Initial visiting of nurses/health officers has no association with health system’s delay, but significantly increases total delay.

General practitioners and nurses/health officers related prolonged delay might be due to low number of trained and experienced professionals in the study area. A recent study in Ethiopia showed that 1/3rd and nearly half of health care workers of public health facilities had poor knowledge and unsatisfactory practice on tuberculosis infection control, respectively [38]. In Ethiopia, Temesgen et al. [39] also showed that more than 80% of healthcare professionals were not trained on tuberculosis infection control. Moreover, a health care provider not specialized in TB [37] and healthcare workers’ poor attitude towards potential patients [40] were found to be among the most influential factors behind the health system’s delay. Thus, further research on level of attitude, experience, knowledge and practice of health care professionals, and providing TB related training of all health professionals is warranted in the study area.

However, the length of health system’s delay was significantly reduced with use of chest X-ray for TB diagnosis as compared to microscopy. This is comparable with reports of Chen et al. [37] in Taiwan with 17% of patients diagnosed based on symptoms and chest X-ray have zero days of health system delay [41]. Our study also showed that use of gene-Xpert and a combination of chest X-ray and microscopy significantly increased total delay (Table 4). This could be explained as gene-Xpert and microscopy requires more processing time than chest X-ray. Additionally, gene-Xpert machines are available only in a few number of government health facilities, which requires more sample and result transportation time especially for remote health facilities. However, further well designed study is needed to assess the contribution of different TB diagnostic tests for health system’s delay.

TB patients who visited private health facilities were 0.5 times less likely to delay for taking treatment as compared to who visited government health facilities. This is in opposition to other previous reports [9, 10] with initial visit of private health facilities found to be an independent predictor of longer delay. It is also in contradiction of previous study in Taiwan that an initial visit to a private practitioner is a risk factor for health system’s delay [37]. This could be explained as previously private health facilities were not part of the DOTS program. But, currently most private health facilities were part of the DOTS program so that TB patients have a chance to start treatment immediately after diagnosis and then transferred to government health facilities for completion of their treatment. Moreover, most private health facilities prefer chest X-ray as the main TB diagnostic test, and which can reduce health system’s delay by 32% (Table 4).

The current study has some limitations. First, we have included patients who came to the DOTS clinics. However, TB patients who didn’t report to a health facility might have experienced longer delay, resulted in the possible underestimation of the observed length of delay. Thus, it is difficult to generalize the result to all TB patients in the study area. Secondly, the duration of patient delay was based on self-report, which might have recall bias as patients may not accurately estimate or remember the exact date of the onset of initial TB symptoms. However, we tried to minimize such bias by collecting data as soon as possible after the patient initiated TB treatment. Additionally, national holidays, religious days, and dates of some events were used for the sake of remembering of events. Since there is no still agreed standard definition of delays in the diagnosis and treatment of TB, we used median value as cut-off. This may limit its comparability with national and international expected delay periods of TB control program, and thus interpretations should be made cautiously.

## Conclusions

In this study, the median patients’, health system’s and total delay was 30 days, 6 days and 36 days, respectively. The late patient health seeking behavior was the major contributor of total delay. About 35.1, 41.1 and 47.4% of patients had prolonged health system’s, patients’ and total delay, respectively. Older age, having larger family size in a house hold, practicing self-medication, SNPTB and EPTB were significant predictors of prolonged patients’ delay. Initial visit of general practitioners and multiple healthcare visits were significant predictors of prolonged health system’s delay. However, lower level of education and use of chest X-ray for TB diagnosis significantly reduced health system’s delay.

Our result indicates the need of immediate interventions that may reduce patients’ delay through communication and social mobilization efforts targeted at informing the general population to seek early health care services when they experience symptoms suggestive of TB. We also recommend that the regional TB program should strengthen the educational campaigns designed to raise public awareness about the signs and symptoms of TB, availability of free services and consequences of early undiagnosed TB disease. In addition, health system strengthening towards TB diagnosis and management must be emphasized through regular training and re-training of all healthcare providers.

## Abbreviations

AFB: Acid fast bacilli
AOR: Adjusted odds ratio
CI: Confidence interval
COR: Crude odds ratio
DOTS: Directly observed treatment short course
EPTB: Extrapulmonary tuberculosis
HIV: Human immunodeficiency virus
IQR: Interquartile ranges
SNPTB: Smear negative pulmonary tuberculosis
SPPTB: Smear positive pulmonary tuberculosis
TB: Tuberculosis
